# Valuing Insect Pollination Services to Safeguard Crop Pollination in South Africa

**DOI:** 10.3390/insects16121190

**Published:** 2025-11-23

**Authors:** Ruan Veldtman, Willem J. de Lange

**Affiliations:** 1 South African National Biodiversity Institute, Kirstenbosch Research Centre, Private Bag X7, Claremont 7735, South Africa; 2 Conservation Ecology and Entomology, Stellenbosch University, Private Bag X1, Matieland 7602, South Africa; 3 National Institute for Theoretical and Computational Science, Stellenbosch 7602, South Africa; 4 Agricultural Economics, Stellenbosch University, Private Bag X1, Matieland 7602, South Africa; wjdel@sun.ac.za

**Keywords:** agronomy, *Apis mellifera*, conservation, economic valuation, environmental management

## Abstract

A worldwide decline in pollinators, combined with the growing demand for pollinator-dependant crops, is increasingly placing the value chains of these crops at risk. Socio-economic impacts can be significant, especially for those economies dependant on export-oriented fruit industries. The valuation of pollinator services is often used for guiding management and investment decisions and aims to not only safeguard these services, but also to balance the supply of, and demand for, pollination services. Although South Africa has a rich pollinator biodiversity and a well-established managed honey bee industry, the supply of pollination is often outpaced by demand. Here for the first time, we attempt a calculation of the aggregate value of insect crop pollination services in South Africa. Using FAO data for 36 insect-dependent crops, we calculate the aggregate value of insect pollination in South Africa using the production function approach. Results show that in 2018, insect pollination was worth over USD 1500 million, equal to 42.2% of the annual production value of insect pollination-dependent crops in South Africa. We highlight the limitations of the valuation in guiding investment in pollination ecosystem services and managed pollination. We suggest that as a good practice, more detailed data related to crop pollination statistics should be collected to allow proper calculation of the ecosystem service contribution to national crop production.

## Simple Summary

Valuing the contribution of insect pollinators can be used to motivate for investment into their conservation and sustainable use. Using South African insect pollination-dependent crops as a case study, we calculate the total value of insect pollination based on how much different crops are dependent on insects to transfer pollen (i.e., little, modest, substantial, and essential). We find that for 2018, insect pollination was worth over USD 1500 million, equal to 42.2% of the annual production value of insect pollination-dependent crops in South Africa. This value cannot, however, be further divided into the contribution of wild versus managed pollinators, even though both groups’ contributions are expected to vary in importance across different crops. We recommend that the renting of managed honey bee colonies for crop pollination is nationally recorded so that at least the managed pollination service can be quantified. We think this is a vital step to effectively invest in the safeguarding of insect pollinators to make insect-dependent crop production more sustainable.

## 1. Introduction

The global supply of insect pollinators struggles to keep pace with the demand for insect pollination services. Not only is the demand for pollination services from prominent pollinators such as honey bees (*Apis mellifera*) growing [1,2], but it has also been well established that the abundance of pollinators is declining [3,4,5]. Both these factors increase the gap between the supply and the demand for pollination services on a global scale. Prominent supply-side factors include habitat loss, climate change, biological invasions, disease, and pesticide use [6,7], whereas changes in global consumer patterns demand more pollination-dependent crops/products [5,8].

Valuation of crop pollination can help make a case for investment into maintaining insect pollination services [9]. Both wild and managed insect pollinators provide pollination services [1,10,11,12]. The value of crop pollination is typically used to justify insect conservation or investment in the keeping of managed pollinators such as honey bees [1,9,13,14,15]. Wild pollinators must be resident to the area to provide pollination services, while managed pollinators can be brought to the service area to perform their pollination service [16]. As a rule, orchards making exclusive use of wild pollination services should avoid large-scale continuous plantings and provide alternative flower resources for resident (i.e., wild) pollinators [17,18]. Carvalheiro et al. [19,20] showed how flower visitation from wild pollinators is negatively affected by distance from natural areas, but how floral-rich corridors could ameliorate some of these impacts. On the other hand, managed pollinators require ample forage throughout the year, which will vary of time and space [21]. Typically, initiatives to promote managed honey bees have targeted large-scale planting of forage species [13]. For example, De Lange et al. [21] illustrated how valuing the forage service provided by alien *Eucalyptus caldocalyx* trees for the maintenance of managed honey bees in the Western Cape of South Africa could be used to guide environmental policy and resource management to safeguard forage availability for the industry.

We provide a first estimate of the value of insect-mediated crop pollination in South Africa on an aggregated scale using a modified proportional total production value (PTPV) approach. It is not intended to be a fully inclusive valuation at this stage, but rather an estimate of the value at risk for vulnerable industries (i.e., those dependent on insect pollination) if forage provision declines. Following the valuation, we discuss whether the value can adequately guide investment in the conservation and management of wild and managed pollinators and associated forage to increase the resilience of the pollination services they provide.

Although most valuation studies are partial in nature, they become informative only when the same set of assumptions are being used when comparing the relative values of services and subsequent pollinator management options. The underlying assumptions also determine, of course, the associated confidence in results. This implies the need for sensitivity analysis to at least show the resulting upper and lower levels of value estimates when the assumptions are varied. Furthermore, economic valuations typically measure values in monetary figures, which is often criticized as being too simplistic as it forces the diversity of value associated with ecosystem services into a single dimension, i.e., monetary value. Nonetheless, valuation is still broadly applied to inform and guide decisions [22,23].

## 2. Materials and Methods

We followed the proportional total production value (PTPV) of Allsopp et al. [24], which requires total annual production, producer market price, and the insect pollination dependence ratios to be known. This method was also commonly used in the GEF Global Pollination Project that spanned from 2010 to 2015, where a standardized template calculated every participating country’s PTPV dependence on pollinators.

The basic formula for the PTPV is as follows:

estimated total production value (TPV) due to insect pollination = crop yield (tons) × price per ton × crop insect dependence factor, or alternatively, the economic value of insect pollinators (EPV) = TPV × insect dependence factor.

Three known shortcomings associated with PTPV method include the following:

- It assumes pollination as a critical production factor and consequently attributes the total production value to pollination before applying the crop dependence factor. It discounts production inputs (labour cost) before applying the dependence factor, which leads to an inflated value estimate for pollination [Remedying this would involve following a residual value approach after the dependence factors have been applied].
- It assumes infinitely high demand elasticities, meaning that decreases in production would not affect market prices. Consumers are consequently forced to seek substitute products. However, not all crops are indeed substitutable, and it is highly likely that farmers would respond to the pollination challenge since doing so could provide them with a competitive advantage over their competition. The resulting decrease in production would not be as severe as been assumed.
- It ignores a possible replacement for insect pollination. Arguably there is no instant replacement for insect pollination due to current labour costs, logistic limitations (thousands of flowers must be pollinated at the same time over a short period), and the lack of any tested method. Replacements can thus be said to be difficult but not impossible [24].

Regardless of these shortcomings the PTPV method employs readily available data of national-scale crop dependency, hectares under cultivation, and market prices. Hence, the same method could be followed for most countries [9]; see, for example, Alebachew [25].

Two assumptions were made for the current study: (i) Partenocarpic crops were excluded from the assessment. Most importantly here, although oranges are listed as having a 5% dependence on insect pollination [1], locally (in South Africa), all varieties are completely partenocarpic and were consequently omitted from the assessment. (ii) Mean dependence ratios were used for crops even though it ignores variation between cultivars, but in the absence of more complete cultivar-level data, there was no alternative. Allsopp et al. [24] noted that the extent of such variance is significant in South Africa. Melathopoulos et al. [15] also confirmed this as a global challenge.

Following Klein et al. [1], we used FAO crop production data for 2018 (the most recent complete South African data available [26] (http//faostat.fao.org, accessed on 20 July 2023)) from 36 crops comprising fruits, oilseeds, pulses, spices, tree nuts, and vegetables. Insect dependence factors were taken from Klein et al. [1] (see Table 1). In some cases, the level of insect dependence showed mixed results for a particular crop type [1], and even if production and price data was available, the data could not be used for this valuation. Data for macadamias and blueberries were obtained from alternative sources [27,28,29] as these are fast becoming important crops for South Africa and were not covered by the FAO data for 2018.

## 3. Results

**Figure 1:**
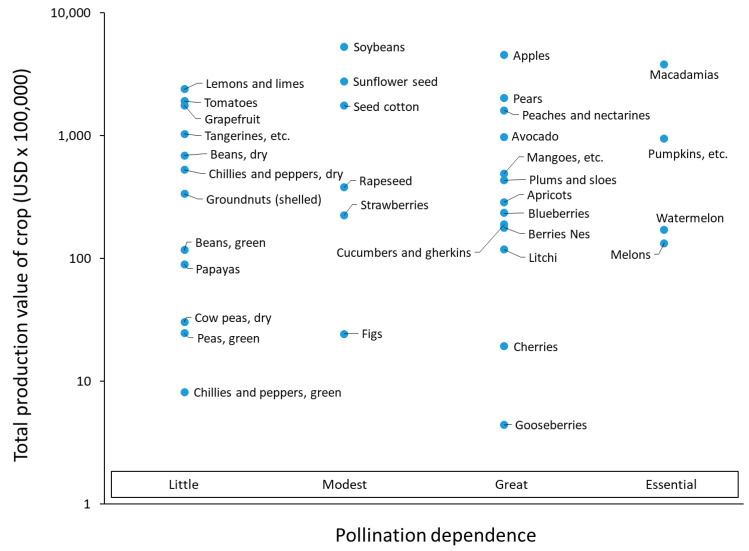
Total production value (TPV) of 36 crops for 2018 as calculated from the FAO data and supplemental resources relative to their insect dependence (see Klein et al., 2007 [1]). Quinces not shown because TPV is less than USD 100,000.

**Figure 2:**
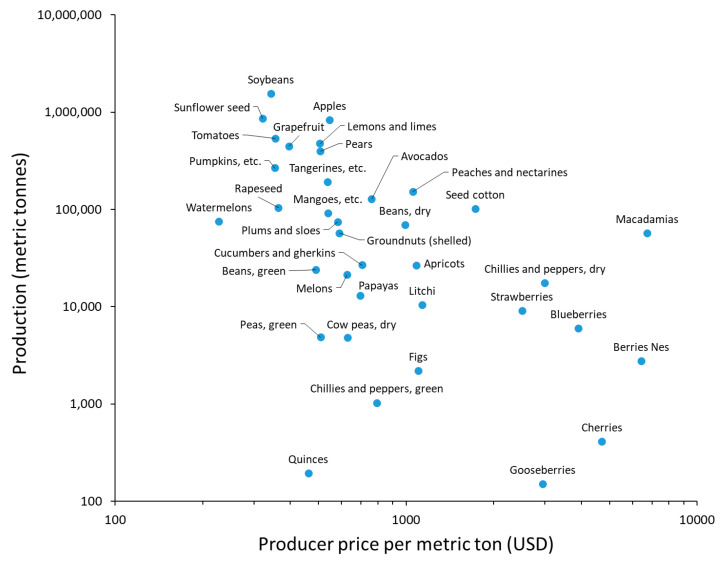
Crop types produced in South Africa in terms of producer price per ton and annual production (36 crops for 2018 as calculated from the FAO data and supplemental resources).

Calculating the annual production value of crops (production multiplied by producer price) revealed that lemons and limes, soybeans, apples, and macadamias have the highest values, respectively, for crops with little, modest, great, and essential pollination dependence on insects (Figure 1). Soybeans had the highest production in 2018, while watermelons fetched the lowest producer price and macadamias the highest (Figure 2).

The annual value of insect pollination in South Africa estimated for 2018 is USD 1,507,276,543 or 42.4% of the total annual production value of insect pollination-dependent crops in South Africa (Table 2). Fruit represented 49.2% of this value, while oilseed crops represented 17.1%, tree nuts 24.0%, and vegetables 9.4%, and pulse and spices had minor contributions of 0.2% and 0.2%, respectively. Although we present only values for 2018, we think this is representative of the larger issues pertaining to South Africa. Interpreting these figures must be carried out in terms of the scale of production, market prices, and insect pollination dependence. There are substitution effects between all three variables, and it could easily be the case that a crop with low insect pollination dependence could be valued high simply because of the scale of production and/or high market prices, thereby overshadowing crops with high insect pollination dependence but with fewer hectares under production and/or lower prices. For example, soybeans (14.92% of TPV) had a high EVP despite a low pollinator dependence simply because of the scale of production.

We were, however, not able to distinguish between the contribution of wild pollinators versus managed honey bees. The only information we could link to the economic value of insect pollination for the highest-ranked crops was whether managed honey bees are rented for pollination or not. Of these crops, only growers of soybeans, sunflower seed, and seed cotton did not rent managed honey bee colonies. Although the top ten crops (according to EPV) that do make use of managed pollinator rental constituted more than 71% of EPV, there is no further quantitative data for more nuanced agronomic calculations. Therefore, one cannot break down the proportional use of managed honey bees per crop, but equally, one cannot assume that managed pollination does not make a major contribution to the value of crop production.

A ranking of the top ten crops by production, total production value, and economic value of insect pollination is shown in Table 3. Macadamias alone contributed USD 361,713,593 or 23.99% of the economic value of insect pollinators. The next highest value was apples with 19.53%.

## 4. Discussion

Valuation methods for pollination typically estimate the value that would be lost due to a loss of pollinators providing such services [18,23]. While often overly conservative by using hive rental cost as a proxy for the value of pollination services, valuation of pollination services mostly fails to adequately motivate for the conservation of wild pollinators specifically [14,24]. Melathopoulos et al. [15] argue that the confidence in the accuracy of the ‘Insect Pollination Economic Value’ is indeed limited compared to biological data (see also [9]). Furthermore, due to the lack of local data on stocking rate recommendations, rates from studies performed in the Northern Hemisphere are often used [24]. In most cases this already predetermines which methods can be used to value insect crop pollination.

The inability to distinguish between the respective share of wild and managed pollination services is not simply a failed academic exercise. This means the value of insect crop pollination services cannot be partitioned between the managed pollination service provided by beekeepers and the ecosystem service provided by wild pollinators. For example, a recent study in the most important apple production area in South Africa could not distinguish between wild and managed honey bees, and as a result, it could not quantify the relative contribution of the pollination ecosystem service to the managed pollinator rental service [30]. This is a general shortcoming of studies valuing insect crop pollination [15,31] and in most cases, the relative contribution of both wild and managed pollinators remains unknown [15]. Allsopp et al. [24] tried to overcome this limitation by using the difference between the expected hive stocking density of managed honey bee hives in deciduous fruit and the declared use by beekeepers to calculate the proportional contribution of wild pollinators. However, their results indicated that wild pollinators contributed approximately 60% of the pollination service, which does not correspond to field data collected on insect pollinator visits [32,33], which found that managed honey bees supply more than 90% of pollination services for several key crops.

We are aware of some studies that have used natural habitat availability as a proxy for the potential pollination service of wild pollinators [34]. The scale of national crop distribution mapping is too high for detailed modelling and mapping of pollination ecosystem services from wild pollinators (as was performed globally [34,35] or as a spatially explicit ecosystem modelling approach [36]). The points made by Melathopoulos et al. (Assumption 2, pp. 64–66 [15]) thus apply: estimations of managed versus wild service contribution could become quite inaccurate.

Unfortunately, there is no information on managed honey bee rentals at the crop level. For example, the hive stocking density advised by the Deciduous Fruit Growers Association was shown by Allsopp et al. [24] to be largely arbitrary and based on very little empirical data. Even if the total hectares of each crop were known, it is still not sufficiently accurate to simply multiply crop hectares with the recommended stocking densities for that crop, as was performed in Allsopp et al. [24] (see also Breeze et al. [37]). The most accurate method would be to annually record the actual number of crop hectares and the number of hives used for the pollination season of each crop. This is quite a logistical undertaking for any country. However, such data is near impossible to collect from South African beekeepers via questionnaires because a substantial proportion of these beekeepers are not affiliated with a body or association [38,39]. Pollination rentals are currently also not recorded by growers of various crop industries, although they do pay for this service, and it would thus be relatively easy to collect this statistic and then total it per crop. We think further effort in this point could potentially solve our national data problem.

In the absence of data on the precise number of managed honey bee hives rented annually for crop pollination to calculate the managed pollination service, a reciprocal estimate of the contribution of wild pollinators is also impossible [24]. However, from a previous review and commentary on South African pollination services, it seems that managed honey bees (not an ecosystem service) provide the bulk of crop pollination services in South Africa [16,32]. Crops like deciduous fruit, blueberries, macadamias, sunflowers, etc., are grown extensively under conventional farming practices (large monocultures, large distance from natural areas, and conventional use of crop protection chemicals) that are not conducive to supporting effective wild pollination services [19].

Mirroring global trends, the overall demand for pollination services is also expected to increase in South Africa. Given the mentioned limitations in the supply of pollination services in South Africa, it is expected that the price of these services will increase, which could initiate a gradual shift of pollination services from lower to higher value crops if the supply of pollination services remains constant. Such a shift can, depending on the cost structure of the relevant crop, lead to structural changes in the South African agricultural sector when, for example, hectares under lower-value-per-hectare crops are replaced by hectares under higher-value-per-hectare crops because of higher levels of affordability of the latter. The crop pollination dependence factor of these crops will determine the hectare substitution ratio (e.g., apple-to-blueberry substitution ratio) and consequently the tempo of such structural change, which evidently is already taking place in the macadamia and blueberry industries of South Africa.

It is worth mentioning that South Africa is currently the largest producer of macadamias in the world with the industry still growing. Hectares under production increased from 34,500 ha in 2012 to approximately 44,775 ha in production in 2019 [28,29], requiring, at four hives per hectare, an astounding 179,100 managed honey bee hives. As macadamias have an insect pollination dependence factor of 95%, a shortage in insect pollinators is likely to occur if the industry continues to grow and if the supply of pollinators does not keep pace. Wild pollinators could help close the looming shortage if standard macadamia cultivation practice is changed to limit the maximum distance from the middle of an orchard to the nearest natural corridor to 300 m [19,20]. This implies a maximum orchard/block size of 9 hectares between natural corridors.

Blueberries is another growing “pollinator-heavy” industry in South Africa with hectares under production almost doubling from 2017 to 2020 [27]. Blueberries are grown under highly intensive conditions largely dependent on managed honey bees, with recommended stocking densities as high as eight hives per hectare and conditions also generally unfriendly to wild pollinators. Blueberries also flower at the same time as early plum cultivars, which means that no substitution (of rental hives) from the deciduous fruit industry will be possible during flowering. It is therefore highly likely that the demand for pollination services will increase more than what current managed pollinators can supply, thereby warning of a potential increased shortage of pollination services.

We therefore stress, based on our national valuation exercise, that increasing the supply of wild pollinators and managed honey bee hives needs to be prioritized to support the resilience of not only pollination services, but also the growth of upcoming industries such as macadamias and blueberries. A cognisant effort to increase natural forage areas within cropping areas will facilitate a natural increase in the supply of wild pollinators. Incidentally, this is the recommendation from the international peer-reviewed literature [11,18]. Such efforts do not imply that vast amounts of land should be taken out of production, neither should they impede natural sanctuaries for pollinators not found in disturbed habitats [40]. Crop cultivation practice should be adjusted and changed in accordance with sustainable agriculture guidelines to account for pollinator-related needs [41,42,43]. Finally, the beekeeping industry in South Africa is in dire need of better self-organization, which will result in improved data collection on the number of managed hives used for crop pollination. This will support more accurate valuation of pollination services provided by beekeepers and consequently an argument for improving forage provisioning of their managed honey bee colonies.

## 5. Conclusions

We propose that our goal to attribute crop pollination to either wild or managed pollination services will help directed investment in the correct ecosystem services that benefit people nationally. This will be the case for any country where insect pollination services are important and require investment and protection. We therefore believe that using such an basic pollination valuation exercise can benefit national policy, provided there is intent and investment to collect relevant data.

## Figures and Tables

**Table 1:** List of crops and insect dependence factors employed in this valuation (36 crops).

Crop	Crop Species	Crop Category (Following FAO)	Average Dependence Factor	Production 2018 (Metric Tonnes)	Producer Price 2018 (USD)
Apples	*Malus domestica*	Fruits	0.65	829,636	545.8
Apricots	*Prunus armeniaca*	Fruits	0.65	26,512	1087.7
Avocados	*Persea americana*	Fruits	0.65	127,568	762.5
Beans, dry	*Phaseolus lunatus*, *P. angularis*, *P. aureus*, *P. mungo*, *P. coccineus*, *P. calcaratus*, *P. aconitifolius*, *P. acutifolius*	Pulse	0.05	69,360	992.2
Beans, green	*Vigna spp.*, *V. unguiculata*, *V. subterranean (syn. Voandzeia subterranea)*, *Phaseolus* spp.	Vegetables	0.05	23,999	489.7
Berries Nes	*Rubus fruiticosus*, *R. chamaemorus*, *R. flagellaris*, *R. trivalis*	Fruits	0.65	2763	6439.2
Blueberries	*Vaccinium corymbosum*, *V. angustifolium*, *V. ashei*, *V. myrtillus*	Fruits	0.65	6000	3909
Cherries	*Prunus cerasus*, *P. avium*	Fruits	0.65	410	4709.1
Chillies and peppers, dry	*Capscium annuum*, *C. frutescens*	Spices	0.05	17,582	3000
Chillies and peppers, green	*Capscium annuum*, *C. frutescens*	Vegetables	0.05	1027	794.7
Cow peas, dry	*Vigna unguiculata*	Vegetables	0.05	4821	631
Cucumbers and gherkins	*Cucumis sativus*	Vegetables	0.65	26,767	707
Figs	*Ficus carica*	Fruits	0.25	2197	1104.2
Gooseberries	*Physalis peruviana*	Fruits	0.65	150	2954.6
Grapefruit	*Citrus grandis*, *C. maxima*, *C. paradisi*,	Fruits	0.05	445,385	396.2
Groundnuts (shelled)	*Arachis hypogaea*	Oil crops	0.05	57,000	590.2
Lemons and limes	*Citrus aurantifolia*, *C. limetta*, *C. limon*	Fruits	0.05	474,149	505.9
Litchi	*Litchi chinensis*	Fruits	0.65	10,400	1136.3
Macadamias	*Macadamia intergrifolia*	Tree nuts	0.95	56,550	6733
Mangoes, mangosteens, and guavas	*Mangifera indica*	Fruits	0.65	91,261	539.1
Melons	*Cucumis melo*	Vegetables	0.95	21,247	627
Papayas	*Carica papaya*	Fruits	0.05	12,909	696.7
Peaches and nectarines	*Prunus persica*, *Persica laevis*	Fruits	0.65	152,444	1055.1
Pears	*Pyrus communis*	Fruits	0.65	397,555	507.7
Peas, green	*Pisum sativum*	Vegetables	0.05	4832	509.4
Plums and sloes	*Prunus domestica*, *P. spinosa*	Fruits	0.65	74,254	583
Pumpkins, squash, and gourds	*Cucurbita maxima*, *C. mixta*, *C. moschata*, *C. pepo*	Vegetables	0.95	266,746	354.4
Quinces	*Cydonia oblonga*, *C. vulgaris*, *C. japonica*	Fruits	0.65	194	462.1
Rapeseed	*Brassica napus*, *B. alba*, *B. hirta*, *Sinapis alba*, *B. nigra*	Oil crops	0.25	103,950	364.73
Seed cotton	*Gossypium hirsutum*, *G. barbadense*, *G. arboreum*, *G. herbaceum*	Oil crops	0.25	101,741	1733.33
Soybeans	*Glycine max*, *G. soja*	Oil crops	0.25	1,540,000	344.2
Strawberries	*Fragaria* spp.	Fruits	0.25	9008	2504
Sunflower seed	*Helianthus annuus*	Oil crops	0.25	862,000	321.6
Tangerines, mandarins, and clementines	*Citrus reticulata*, *C. unshiu*	Fruits	0.05	191,866	538.3
Tomatoes	*Lycopersicon esculentum*	Vegetables	0.05	537,257	355.3
Watermelons	*Citrullus lanatus*	Vegetables	0.95	75,160	227.9

**Table 2:** Annual value (in millions of US dollars) of insect pollination for South African crop types in 2018.

Crop Category	Average Value per Metric Ton	Total Production Value (TPV)	Economic Value of Insect Pollinators (EVP)	Ratio of Vulnerability
	USD	Mil USD	Mil USD	%
Fruits	576	1643.21	740.93	45.1%
Oil crops	396	1055.19	257.07	24.4%
Pulse	992	68.82	3.44	5.0%
Spices	3000	52.75	2.64	5.0%
Tree nuts	6733	380.75	361.71	95.0%
Vegetables	367	352.87	141.49	40.1%
Total		3553.59	1507.28	42.4%

**Table 3:** Top 10 crops in South Africa relative to production, total production value (TPV), and economic value of insect pollinators (EVP) in 2018 (% indicates contribution to total). For EVP-ranked crops it is indicated whether managed honey bee colonies are rented for pollination (based on observations for South African agriculture industry).

Rank	Production	%	TPV	%	EVP	%	Hives Rented
1	Soybeans	23.25	Soybeans	14.92	Macadamias	23.99	Yes
2	Sunflower seed	13.01	Apples	12.74	Apples	19.53	Yes
3	Apples	12.52	Macadamia	10.71	Soybeans	8.79	No
4	Tomatoes	8.11	Sunflower seed	7.80	Pears	8.70	Yes
5	Lemons and limes	7.16	Lemons and limes	6.75	Peaches and nec.	6.94	Yes
6	Grapefruit	6.72	Pears	5.68	Pumpkins, etc.	5.96	Yes
7	Pears	6.00	Tomatoes	5.37	Sunflower seed	4.60	No
8	Pumpkins, etc.	4.03	Grapefruit	4.97	Avocados	4.19	Yes
9	Tangerines, etc.	2.90	Seed cotton	4.96	Seed cotton	2.92	No
10	Peaches and nec.	2.30	Peaches and nec.	2.94	Mangoes, etc.	2.12	Yes

Total production = 6,624,700 tons; TPV = USD 3,553,589,886; EPV = USD 1,507,276,371.

## Data Availability

The original contributions presented in this study are included in the article. Further inquiries can be directed to the corresponding author.
